# Associations between prediagnostic aspirin use and ovarian tumor gene expression

**DOI:** 10.1002/cam4.6386

**Published:** 2023-08-01

**Authors:** Naoko Sasamoto, Paul A. Stewart, Tianyi Wang, Zachary J. Thompson, Sean J. Yoder, Jonathan L. Hecht, John L. Cleveland, Jose Conejo‐Garcia, Brooke L. Fridley, Kathryn L. Terry, Shelley S. Tworoger

**Affiliations:** ^1^ Department of Obstetrics and Gynecology Brigham and Women's Hospital and Harvard Medical School Boston Massachusetts USA; ^2^ Department of Biostatistics and Bioinformatics H. Lee Moffitt Cancer Center and Research Institute Tampa Florida USA; ^3^ Department of Cancer Epidemiology H. Lee Moffitt Cancer Center and Research Institute Tampa Florida USA; ^4^ Molecular Genomics Core Facility H. Lee Moffitt Cancer Center and Research Institute Tampa Florida USA; ^5^ Department of Pathology Beth Israel Deaconess Medical Center and Harvard Medical School Boston Massachusetts USA; ^6^ Department of Tumor Biology H. Lee Moffitt Cancer Center and Research Institute Tampa Florida USA; ^7^ Department of Immunology H. Lee Moffitt Cancer Center and Research Institute Tampa Florida USA; ^8^ Department of Epidemiology Harvard T.H. Chan School of Public Health Boston Massachusetts USA

**Keywords:** aspirin, gene expression, interferon gamma, ovarian cancer, tumor microenvironment

## Abstract

**Background:**

Aspirin use has been associated with reduced ovarian cancer risk, yet the underlying biological mechanisms are not fully understood. To gain mechanistic insights, we assessed the association between prediagnosis low and regular‐dose aspirin use and gene expression profiles in ovarian tumors.

**Methods:**

RNA sequencing was performed on high‐grade serous, poorly differentiated, and high‐grade endometrioid ovarian cancer tumors from the Nurses' Health Study (NHS), NHSII, and New England Case–Control Study (*n* = 92 cases for low, 153 cases for regular‐dose aspirin). Linear regression identified differentially expressed genes associated with aspirin use, adjusted for birth decade and cohort. False discovery rates (FDR) were used to account for multiple testing and gene set enrichment analysis was used to identify biological pathways.

**Results:**

No individual genes were significantly differentially expressed in ovarian tumors in low or regular‐dose aspirin users accounting for multiple comparisons. However, current versus never use of low‐dose aspirin was associated with upregulation of immune pathways (e.g., allograft rejection, FDR = 5.8 × 10^−10^; interferon‐gamma response, FDR = 2.0 × 10^−4^) and downregulation of estrogen response pathways (e.g., estrogen response late, FDR = 4.9 × 10^−8^). Ovarian tumors from current regular aspirin users versus never users were also associated with upregulation in interferon pathways (FDR <1.5 × 10^−4^) and downregulation of multiple extracellular matrix (ECM) architecture pathways (e.g., ECM organization, 4.7 × 10^−8^).

**Conclusion:**

Our results suggest low and regular‐dose aspirin may impair ovarian tumorigenesis in part via enhancing adaptive immune response and decreasing metastatic potential supporting the likely differential effects on ovarian carcinogenesis and progression by dose of aspirin.

## Graphical abstract

Aspirin use has been associated with reduced ovarian cancer risk; however, the underlying biological mechanisms are not fully understood. To investigate the potential biological pathways impacted by aspirin use in the ovarian tumor microenvironment, we conducted RNA sequencing on over 200 invasive ovarian tumors from well‐characterized ovarian cancer patients and examined the association between prediagnosis low and regular‐dose aspirin use and ovarian tumor gene expression profiles. Our results show prediagnosis low‐dose aspirin use was associated with antitumor immunity and regular‐dose aspirin use was associated with decreased metastatic potential, suggesting differential effects on ovarian carcinogenesis and progression by dose of aspirin.

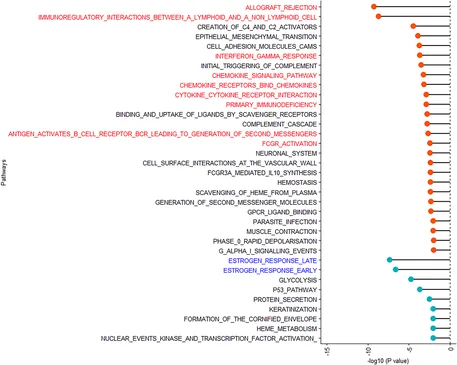

## 1 INTRODUCTION

Increasing evidence supports the role of aspirin use in cancer prevention and survival outcomes.^1^ In ovarian cancer, epidemiological studies have reported that aspirin use is associated with around 10%–20% reduction in ovarian cancer risk,^2^ with a stronger inverse association observed for low‐dose aspirin use.^3^ Furthermore, while studies examining the association between prediagnosis aspirin use and ovarian cancer survival have been null,^4^ aspirin use after diagnosis has been associated with improved ovarian cancer‐specific survival,^5^ although evidence is still limited.^6^

Aspirin reduces inflammation, modulates immune responses, and has antithrombotic effects,^7^ and collectively these effects are thought to reduce initiation and progression of ovarian cancer.^8^ However, the underlying biological mechanism of how aspirin use impacts ovarian carcinogenesis and progression is not fully understood. To gain mechanistic insights, we assessed the association between gene expression profiles in ovarian tumors and current low‐dose and regular‐dose aspirin use in the 1–2 years before diagnosis, with a focus on type II ovarian tumors (i.e., high‐grade serous, poorly differentiated, and high‐grade endometrioid histology).^9^

## 2 MATERIALS AND METHODS

### 2.1 Study populations

#### 2.1.1 Nurses' Health Studies

The Nurses' Health Study (NHS) is a prospective cohort study based in the United States that enrolled 121,000 female nurses aged 30–55 years in 1976 and the Nurses' Health Study II (NHSII) enrolled 116,429 female nurses aged 25–42 in 1989.^10^, ^11^ Participants were followed every 2 years using questionnaires that asked updated exposure data on lifestyle, reproductive factors, and medical history including medication use. If a participant reported an ovarian cancer diagnosis or died of ovarian cancer using the National Death Index, the diagnosis was confirmed using pathology reports or linkage to state cancer registries. A pathologist (J.H.) reviewed the medical and pathology records and abstracted stage information. As described previously, ovarian tumor formalin fixed paraffin‐embedded (FFPE) tissue blocks were collected from these ovarian cancer cases, and slides were centrally reviewed by single gynecologic pathologist to determine histology and grade (J.H.).^12^ There were FFPE blocks available from 209 cases (*n* = 157 in NHS and *n* = 52 in NHSII) diagnosed from 1995 to 2013 with high‐grade serous, poorly differentiated, or high‐grade endometrioid histology. We excluded cases diagnosed before 1995 due to poor quality/quantity of extracted RNA. The study protocol was approved by the Brigham and Women's Hospital and Harvard T.H. Chan School of Public Health Institutional Review Boards, and those of participating registries as required.

#### 2.1.2 New England Case–Control Study

The New England Case–Control Study (NECC) is a population‐based case–control study of ovarian cancer based in New Hampshire and Eastern Massachusetts that enrolled participants aged 18–80 years over three phases (1992–1997, 1998–2002, and 2003–2008).^13^ Briefly, eligible cases were identified using registries of area hospitals and 2203 (71%) participated in the study. Detailed information on exposures including lifestyle, reproductive factors, and medical history including medication use that occurred up to 1 year before diagnosis was collected through in‐person interviews. Ovarian cancer diagnosis was confirmed through surgical and pathological report review and information on stage was abstracted. We collected FFPE ovarian tumor tissue blocks from confirmed ovarian cancer cases and histology and grade was coded with centralized pathology review by a pathologist (J.H.) as previously described.^12^ There were FFPE blocks available from 109 cases with high‐grade serous, poorly differentiated, or high‐grade endometrioid histology diagnosed from 1995 to 2008. The study was approved by the Brigham and Women's Hospital and Dartmouth College Institutional Review Boards.

### 2.2 Assessment of aspirin use

In NHS/NHSII, low‐dose aspirin (<100 mg) and regular‐dose aspirin use were assessed in biennial questionnaires. In NHS, regular‐dose aspirin use of two or more times per week in the prior 2 years was asked in all questionnaires except 1986. In NHSII, the same question was assessed in 1989, 1993, and every 2 years thereafter. In 2000 (NHS) and 2001 (NHSII), specific questions were added about low‐dose aspirin use. Women were defined as current users if they reported using aspirin (regular or low‐dose) in the survey 1 cycle prior to ovarian cancer diagnosis; never users were defined as those women who reported no use of aspirin 2 or more times per week on any questionnaire prior to the diagnosis. In the NECC study, use of any regular‐dose aspirin continuously for 6 months or longer was assessed up to 1 year before diagnosis. Current use was defined as use at 1 year prior to diagnosis and never use was defined as not using aspirin continuously for 6 months or longer at any time prior to diagnosis. Use of low‐dose aspirin was not assessed in the NECC study. Women who were former aspirin users (i.e., used aspirin at a time other than 1–2 years before diagnosis) were excluded due to the potential for misclassification of exposure status (*n* = 43 for NHS, 6 for NHSII, and 11 for NECC for regular‐dose aspirin use; *n* = 10 for NHS, 2 for NHSII for low‐dose aspirin use).

### 2.3 RNA extraction and RNA sequencing

For each case, treatment naïve primary FFPE ovarian tumor blocks were obtained and a 1.5 mm diameter tumor tissue core was isolated from tumor areas circled by the gynecologic pathologist. DNA and RNA were simultaneously extracted using the Qiagen All‐Prep RNA Isolation Kit as described.^14^ In brief, we used the Illumina TruSeq™ RNA Exome Library Preparation Kit (Illumina Inc.) following the manufacturer's protocol to prepare the RNA‐sequencing (RNA‐Seq) libraries and sequenced on the multiple NextSeq 500 High‐output sequencing runs to target about 25 M pairs of 75‐base reads per sample. RNA and DNA were extracted from 323 tumor cores, and of those, 35 (11%) samples that had DV200 values ≤15% were excluded from further analysis. RNA sequencing was conducted on 288 RNA samples (which include four replicate samples) and successfully generated data on 253 samples (*n* = 122 in NHS, *n* = 45 in NHSII, *n* = 86 in NECC). Among these, the current analysis included 92 type II ovarian tumor samples with data on low‐dose aspirin use (NHS/NHSII only) and 153 type II ovarian tumor samples with data on regular‐dose aspirin use (*n* = 126 in NHS/NHSII and *n* = 27 in NECC).

### 2.4 RNA‐Seq data analysis

To filter genes with low expression, we used function *filterByExpr*() from the R package *edgeR*. As a result, 14,483 genes were included in the analysis. To account for differences in library size between samples, Trimmed Mean of M values (TMM) normalization using *calcNormFactors*(), also from *edgeR*, was applied. All data were then transformed using *voom*. Using principal component analyses, we did not observed any batch or study effects.^14^ Thus, we used pooled data from NHS, NHSII, and NECC. Logistic models were used to examine the associations between gene expression and outcomes of interest (i.e., current low‐dose aspirin use vs. never use, current regular‐dose aspirin use vs. never use) using *limma* while adjusting for birth decade and study site. Benjamini–Hochberg's false discovery rate (FDR) was used to account for multiple testing.

To identify biological pathways associated with current low‐dose aspirin use or current regular‐dose aspirin use, gene set enrichment analysis (GSEA) with *fgsea* using the log fold changes from *limma* as the ranks was completed using the Cancer Hallmarks, KEGG, and Reactome gene sets. We calculated the Normalized Enrichment Scores (NES) to enable direct comparison of results across gene sets. Gene sets were considered significant if the FDR < 0.01. Heatmaps were created including individual genes within the significant pathways with an unadjusted *p*‐value <0.10 for current low‐dose aspirin use or current regular‐dose aspirin use using the R library *ComplexHeatmap*.

## 3 RESULTS

Our analysis on low‐dose aspirin use included 33 current users in the 1–2 years before diagnosis and 59 never users (Table 1). In both groups, more than 70% of cases were diagnosed at an advanced stage. Current low‐dose aspirin users were older at diagnosis with median age of 73 years compared with never users whose median age was 63 years. For regular‐dose aspirin use, our analysis included 61 current regular‐dose aspirin users and 92 never users. The majority (>70%) were advanced stage at diagnosis in both current and never regular‐dose aspirin users, with an average age at diagnosis of 68 years for current regular‐dose aspirin users and 57 years for never users. Of the 33 cases who were current low‐dose aspirin users in the 1–2 years prior to diagnosis, 25 (76%) reported as also being current regular‐dose aspirin users. Of the 59 cases who reported never use of low‐dose aspirin, 7 (12%) were current regular‐dose aspirin users.

**TABLE 1:** Clinical characteristics of ovarian cancer cases in NHS, NHSII, and NECC studies by aspirin use status.^a^

	Low‐dose aspirin use		Regular‐dose aspirin use	
	Current	Never	Current	Never
	(*n* = 33)	(*n* = 59)	(*n* = 61)	(*n* = 92)
Calendar year at diagnosis, median (Q1‐Q3)	2007 (2005–2009)	2005 (2002–2008)	2003 (1999–2007)	2004 (2001–2007)
Age at diagnosis, years, median (Q1‐Q3)	73 (62–78)	63 (55–71)	68 (61–75)	57 (50–63)
Study, *n* (%)				
NECC	0 (0%)	0 (0%)	4 (7%)	68 (74%)
NHS	26 (79%)	35 (59%)	50 (82%)	4 (4%)
NHSII	7 (21%)	24 (41%)	7 (11%)	20 (22%)
Stage, *n* (%)				
I	4 (12%)	12 (20%)	9 (15%)	12 (13%)
II	3 (9%)	4 (7%)	6 (10%)	10 (11%)
III	22 (67%)	36 (61%)	38 (62%)	62 (67%)
IV	4 (12%)	6 (10%)	8 (13%)	6 (7%)
Missing	0 (0%)	1 (2%)	0 (0%)	2 (2%)
Histology				
High‐grade serous/poorly differentiated	31 (94%)	48 (81%)	54 (89%)	88 (97%)
High grade endometrioid	2 (6%)	11 (19%)	7 (11%)	3 (3%)

Abbreviations: NHS, Nurses' Health Study; NHSII, Nurses' Health Study II; NECC, New England Case–Control Study; Q, quartile.

^a^ Current use was defined as having used low‐dose aspirin or regular‐dose aspirin 1–2 years prior to ovarian cancer diagnosis, never use was defined as having never regularly used low‐dose aspirin or regular‐dose aspirin prior to ovarian cancer diagnosis.

### 3.1 Ovarian tumor gene expression profiles of current versus never low‐dose aspirin use patients

**FIGURE 1:**
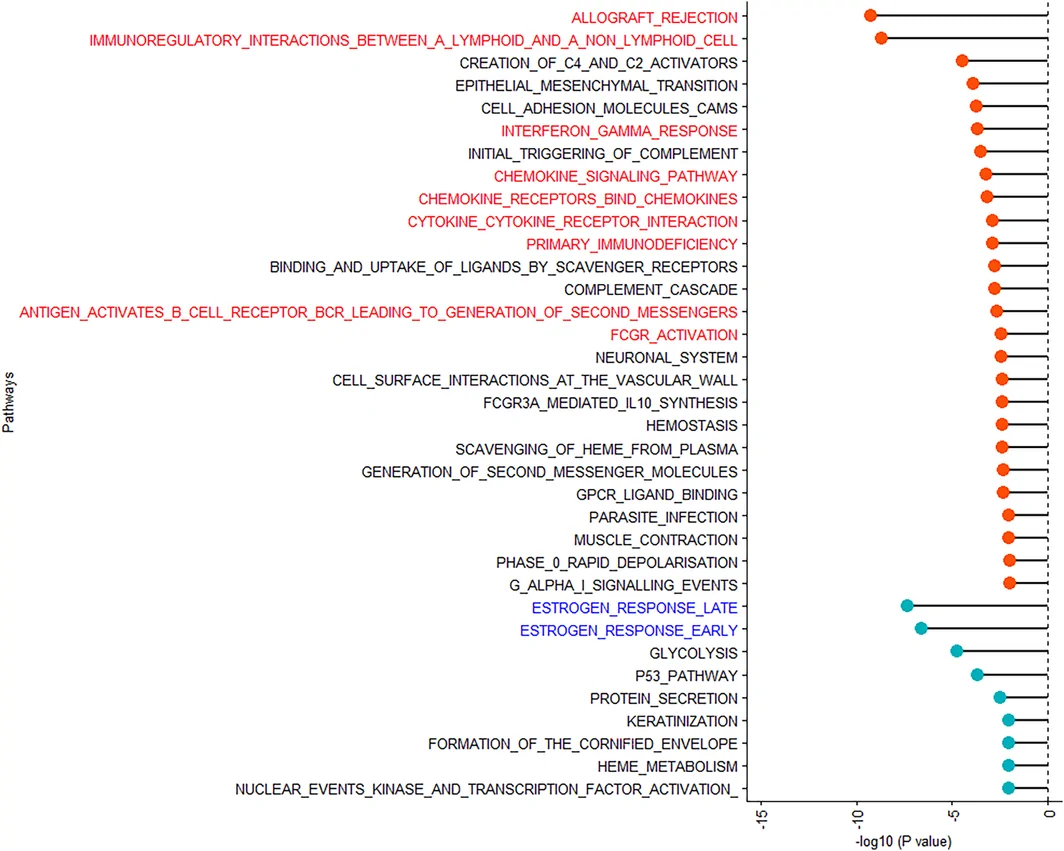
Significant pathways associated with current low‐dose aspirin use in the 1–2 years prior to ovarian cancer diagnosis compared with never low‐dose aspirin use in type II ovarian tumor tissue in Nurses' Health Study (NHS), Nurses' Health Study II (NHSII), and New England Case–Control Study (NECC) (false discovery rate (FDR) <0.01). Pathways associated with current low‐dose aspirin use are presented in the order of statistical significance and FDR plotted on the x‐axis. Upregulated pathways are denoted by red bubbles, and downregulated pathways are denoted by blue bubbles.

When comparing individual gene expression in ovarian tumors between current low‐dose aspirin users and never users, none of the individual gene associations were statistically significant after adjusting for multiple comparisons (FDR > 0.05; Table S1). However, GSEA revealed 35 pathways that were significantly associated with current low‐dose aspirin use compared with never use (FDR <0.01; Figure 1; Table S2). Notably, multiple pathways related to inflammation and immunity were upregulated (FDR *p* < 3.5 × 10^−3^), while two estrogen‐related pathways were downregulated (FDR *p* < 2.6 × 10^−7^) in ovarian tumors of current low‐dose aspirin users compared with never users.

**FIGURE 2:**
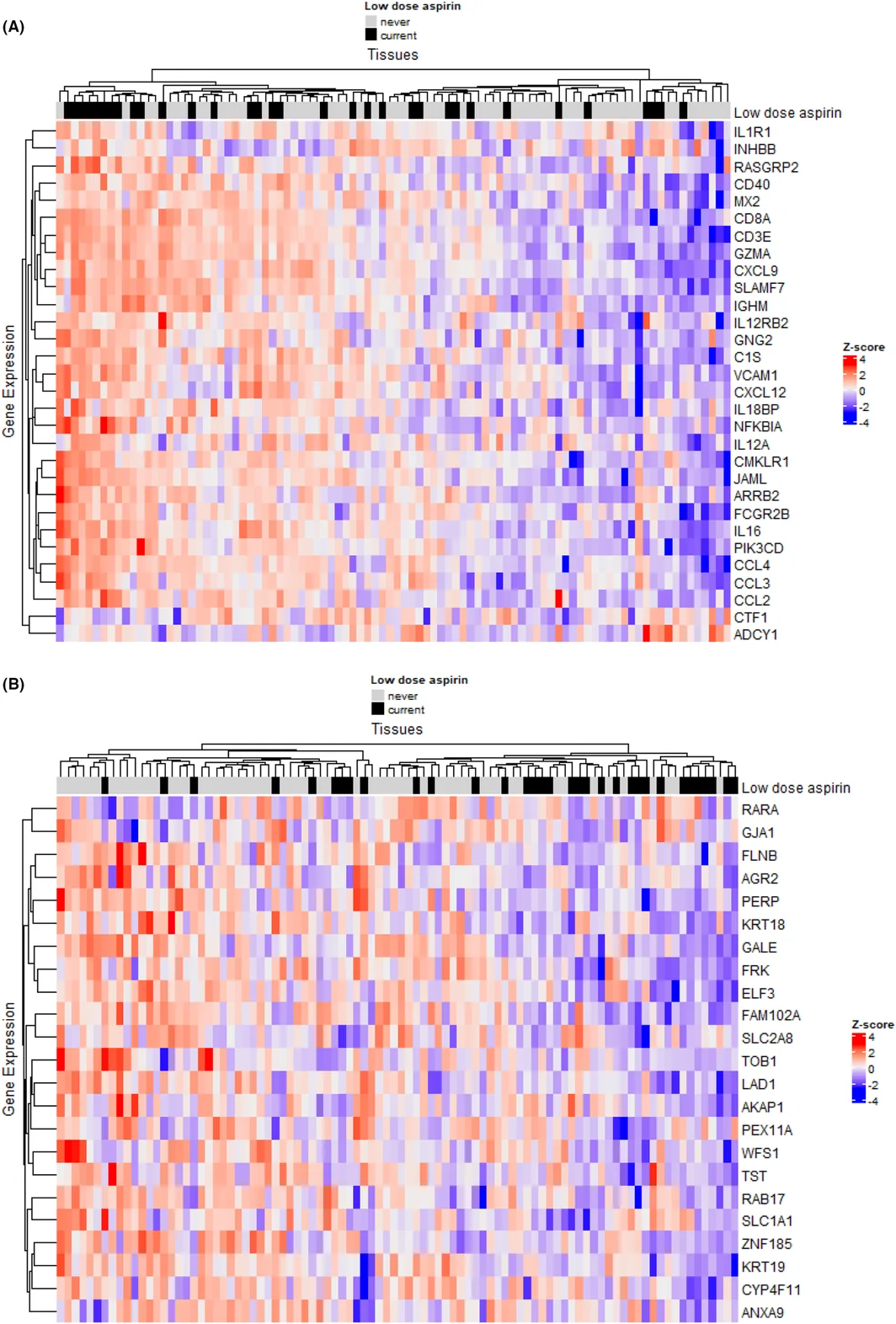
Differentially expressed genes included in significant pathways associated with current versus never low‐dose aspirin use in type II ovarian tumors. (A) Differentially expressed inflammation and immune‐related pathway genes that are associated with current versus never low‐dose aspirin use in type II ovarian tumors. Within the significant pathways (i.e., interferon‐gamma response, chemokine signaling pathway, chemokine receptors bind chemokine, cytokine‐cytokine receptor interaction, allograft rejection, immunoregulatory interactions between a lymphoid and a nonlymphoid cell, primary immunodeficiency, antigen activates B‐cell receptor BCR leading to generation of second messengers, and FCGR activation), individual genes that were associated with current low‐dose aspirin use with unadjusted *p*‐value <0.10 were included in the heatmap. (B) Differentially expressed estrogen‐related pathway genes that are associated with current versus never low‐dose aspirin use in type II ovarian tumors. Within the significant pathways (i.e., estrogen response late and estrogen response early), individual genes associated with current low‐dose aspirin use with unadjusted *p*‐value<0.10 were included in the heatmap.

Since genes overlap across these pathways (Figure S1), we also examined the individual genes that were associated with current low‐dose aspirin use (unadjusted *p* < 0.10) in the significant inflammation and immunity‐related pathways (i.e., interferon‐gamma response, chemokine signaling pathway, chemokine receptors bind chemokine, cytokine‐cytokine receptor interaction, allograft rejection, immunoregulatory interactions between a lymphoid and a nonlymphoid cell, primary immunodeficiency, antigen activates B‐cell receptor BCR leading to generation of second messengers, and FCGR activation; Figure 2A). Interestingly, T‐cell markers (e.g., CD8A and CD3E) and cytokines related to the Th1 immune response (e.g., IL12A and IL12RB2) were upregulated in tumors from current low‐dose aspirin users compared with never users, suggesting augmented immune surveillance. Furthermore, individual genes with an unadjusted *p* < 0.10 in the significant estrogen early and late response pathways included those related to cell proliferation and migration (e.g., AGR2,^15^ ANXA9,^16^ KRT18,^17^ and LAD1^18^), which were downregulated in tumors from current low‐dose aspirin users compared with never users (Figure 2B).

### 3.2 Ovarian tumor gene expression profiles of current versus never regular‐dose aspirin use patients

**FIGURE 3:**
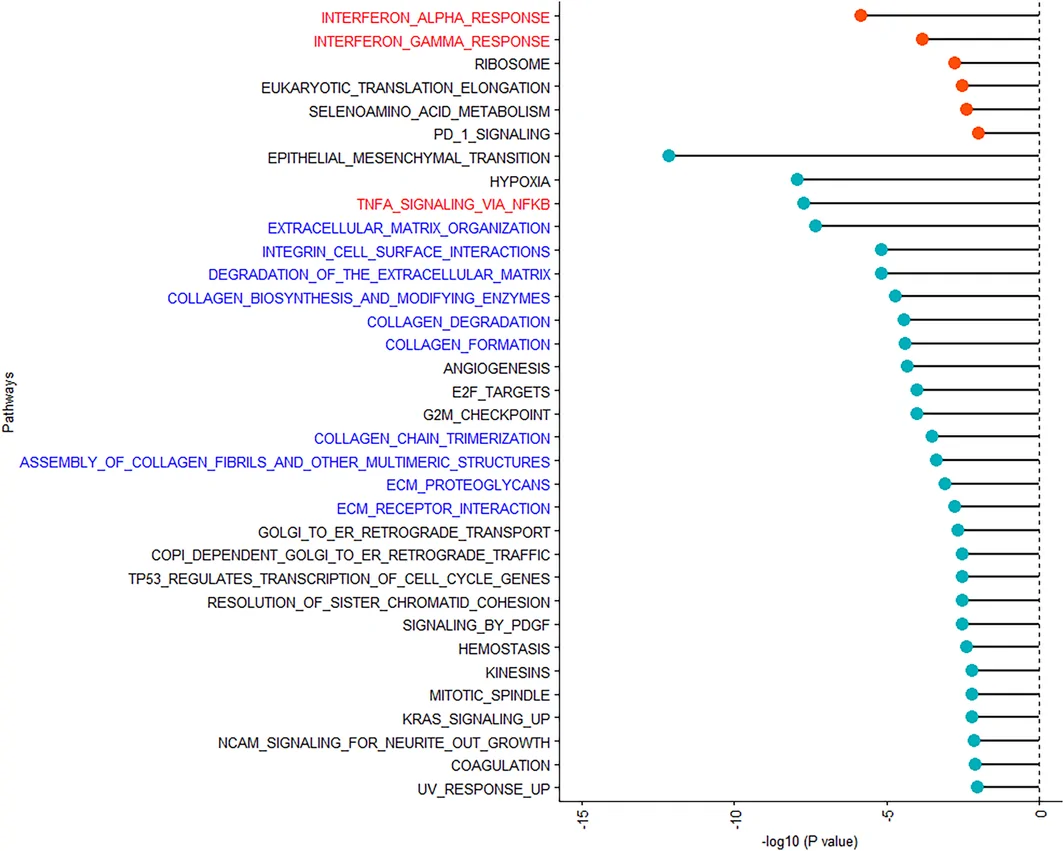
Significant pathways associated with current regular‐dose aspirin use in the 1–2 years prior to ovarian cancer diagnosis compared with never regular‐dose aspirin use in type II ovarian tumor tissue in Nurses' Health Study (NHS), Nurses' Health Study II (NHSII), and New England Case–Control Study (NECC) (false discovery rate (FDR) <0.01). Pathways associated with current regular‐dose aspirin use are presented in the order of statistical significance and FDR plotted on the x‐axis. Upregulated pathways are denoted by red bubbles, and downregulated pathways are denoted by blue bubbles.

**FIGURE 4:**
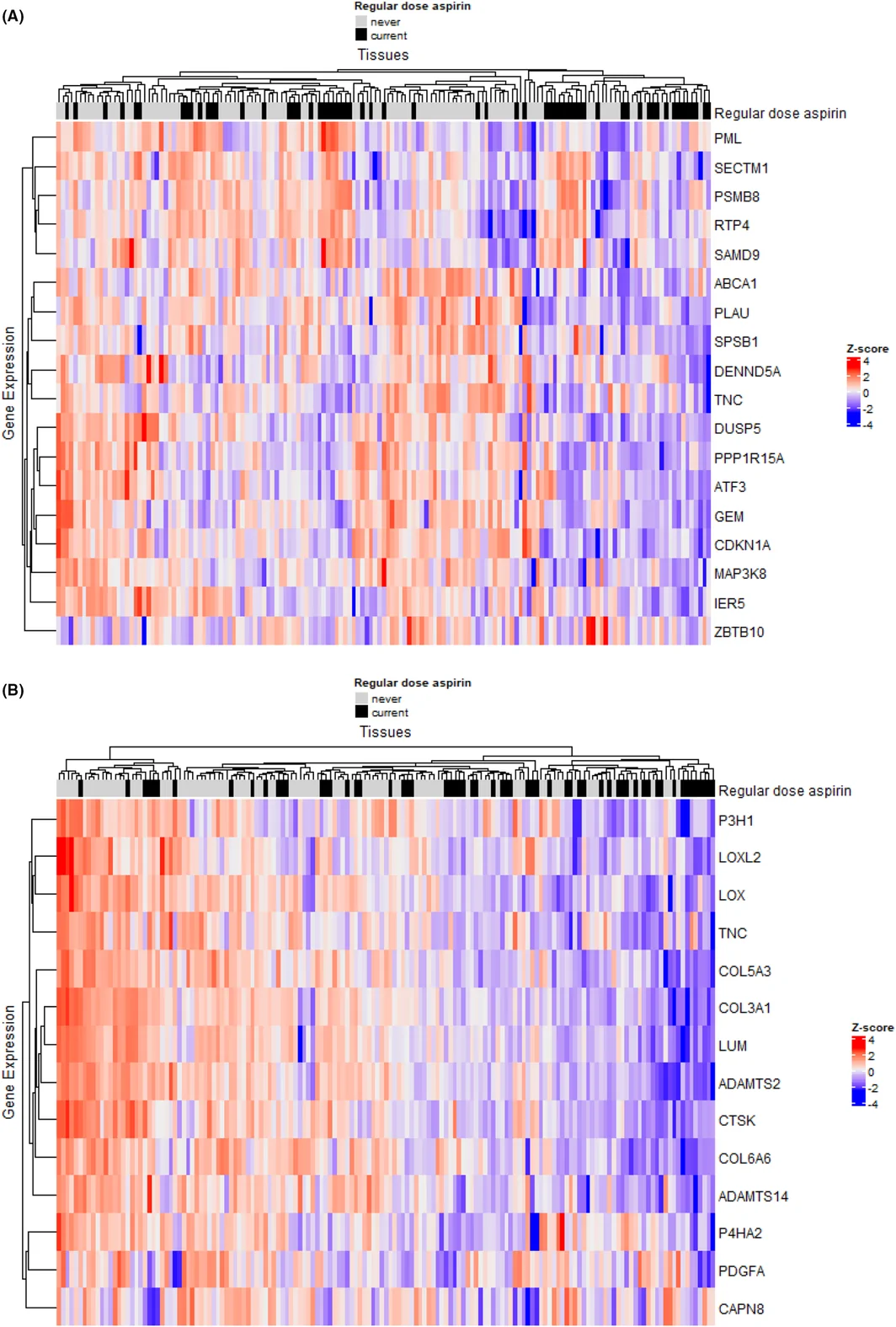
Differentially expressed genes included in significant pathways associated with current versus never regular‐dose aspirin use in type II ovarian tumors. (A) Differentially expressed inflammatory pathway genes that are associated with current versus never regular‐dose aspirin use in type II ovarian tumors. Within the significant pathways (i.e., TNFa signaling via NFkB, interferon alpha response, and interferon‐gamma response), individual genes that are associated with current regular‐dose aspirin use with unadjusted *p*‐value<0.10 were included in the heatmap. (B) Differentially expressed extracellular matrix architecture pathway genes that are associated with current regular‐dose aspirin use versus never aspirin use in type II ovarian tumors. Within the significant pathways (i.e., extracellular matrix organization, integrin cell surface interactions, degradation of the extracellular matrix, collagen biosynthesis and modifying enzymes, collagen degradation, collagen formation, collagen chain trimerization, assembly of collagen fibrils and other multimeric structures, ECM proteoglycans, and ECM receptor interaction), individual genes that were associated with current regular‐dose aspirin use with unadjusted *p*‐value<0.10 were included in the heatmap.

In comparing these cohorts, no individual genes were found to be differentially expressed with statistical significance in tumors from patients who had regular‐dose aspirin use after adjusting for multiple comparisons (FDR >0.05; Table S3). However, 34 biological pathways were significantly associated with current regular‐dose aspirin use compared with never use (FDR <0.01; Figure 3, Table S4). Again, multiple inflammatory pathways were identified, with factors related to interferon response (e.g., interferon alpha response and interferon beta response) being upregulated (FDR <1.5 × 10^−4^) and other inflammatory pathways (e.g., TNFα signaling via NFkB) being downregulated (FDR <2.0 × 10^−8^). Furthermore, multiple pathways related to extracellular matrix architecture (e.g., extracellular matrix organization, integrin cell surface interactions) and collagen structure were downregulated (FDR <1.7 × 10^−3^). When we examined individual genes that were associated with current regular‐dose aspirin use in the significant up‐ and downregulated inflammatory pathways (Figure S2), genes related to the induction of cellular senescence (e.g. PML,^19^ PLAU,^20^ PPP1R15A,^21^ and CDKN1A^22^) and immunosuppression (e.g. ABCA1,^23^, ^24^ and TNC^25^, ^26^) were downregulated in tumors of current regular aspirin users compared with never users (Figure 4A). Conversely, genes related to tumor immune cell infiltration and antigen presentation (e.g., SECTM1,^27^ PSMB8,^28^, ^29^, ^30^ SAMD9,^31^, ^32^ and RTP4^33^, ^34^) were upregulated. Finally, when individual genes included in the significant extracellular matrix architecture pathways were examined, multiple genes related to metastasis (e.g. LOXL2,^35^, ^36^ COL3A1,^37^, ^38^ LUM,^37^ and CTSK^39^) were downregulated in tumors of current regular‐dose aspirin users compared with never users (Figure 4B).

## 4 DISCUSSION

Aspirin use, particularly low‐dose aspirin, is associated with reduced risk and improved survival in ovarian cancer and other cancer types.^1^, ^2^, ^5^ While the underlying mechanism has been postulated to be due to reduced inflammation, our analysis of tumor gene expression profiles in low‐dose and regular‐dose aspirin users revealed the opposite—increased expression of inflammatory and immune cell effectors—along with several other pathways and genes that are affected by aspirin use in the ovarian tumor microenvironment. Prior work has demonstrated that inflammatory cytokines can trigger and enhance an immune response, possibly explaining the unexpected association with higher expression of pro‐inflammatory pathways. Interestingly, when we examined individual genes that were significantly associated with current low‐dose aspirin use in the identified biological pathways, a hallmark of most tumors of current low‐dose aspirin users was upregulation of the cytotoxic/Th1 adaptive immune response, suggesting aspirin use improves immune surveillance in the ovarian tumor microenvironment compared with never users. For example, we observed that IL12A and IL12RB2 being upregulated in ovarian tumors of current low‐dose aspirin users compared with never users, which are inflammatory genes included in the cytokine‐cytokine receptor interaction pathway but are also known to enhance Th1 immune response.^40^ Notably, tumors of current regular‐dose aspirin users were also more likely to have upregulation of genes related to tumor immune cell infiltration and express reduced levels of genes associated with immunosuppression, tumor cell metastasis, and cellular senescence. Strikingly, one major pathway overlapped for users of low versus regular‐dose aspirin, namely upregulation of interferon‐gamma response, suggesting this is a driver of the beneficial effects of aspirin in impairing ovarian carcinogenesis regardless of dose. Given the key roles of interferon‐gamma in cytotoxic/ Th1 antitumor responses, our data support the notion that low‐dose aspirin use reduces ovarian cancer risk by augmenting T‐cell‐mediated immune surveillance.

Our results suggest that low‐dose aspirin upregulates cytotoxic T cell and Th1 (innate) immune responses in the ovarian tumor microenvironment, which are known to be related to improved survival.^41^ Specifically, there was substantial upregulation of IL‐12, primarily expressed in T cells and B cells, that leads to upregulation of interferon‐gamma (IFN‐ɣ), a key link between innate and adaptive immunity.^40^ Notably, the interferon‐gamma response pathway was also significantly upregulated in tumors of women who used low‐dose aspirin. We also observed upregulation of CXCL9 expression, which is induced by IFN‐ɣ, in current low‐dose aspirin users. Elevated CXCL9 expression is associated with improved survival in patients with ovarian cancer.^42^ Furthermore, in animal models, CXCL9 overexpression promotes tumor T‐cell accumulation and improves immune checkpoint blockade therapy response in previously resistant mice.^43^ Interestingly, low‐dose aspirin users also showed upregulation of CD40 in their tumors, which is expressed by dendritic cells and B cells, and upregulation of VCAM1,^44^ which governs leukocyte trafficking. Overall, our results suggest use of low‐dose aspirin in the period before diagnosis leads to increases in the ovarian tumor immune response, which could explain the lower risk and improved survival observed in epidemiologic studies.^2^, ^3^, ^5^, ^45^ Interestingly, genes related to cell proliferation and migration were also downregulated in tumors of current low‐dose aspirin users compared with never users, which is in line with prior retrospective clinical studies reporting the effect of low‐dose aspirin on reducing risk of cancer metastasis in humans and animal models.^46^

In addition to upregulation of IFN‐ɣ and cytotoxic/Th1 response pathways, several other pathways were also associated with current regular‐dose aspirin use. Multiple pathways related to extracellular matrix architecture were significantly downregulated in tumors of current regular aspirin users, specifically pathways related to collagen formation, biosynthesis, trimerization, assembly, and degradation. In line with these observations, in animal models aspirin has been shown to reduce collagen deposition and inhibit liver tumor growth,^47^ an effect that is consistent with aspirin's anticoagulant action, including effects on heparinase^48^ and collagen‐induced coagulation.^49^

Genes related to cellular senescence were also downregulated in tumors of current regular‐dose aspirin users compared with never users, although the mechanism of how aspirin impacts cellular senescence in experimental models is not fully determined.^50^ Further research is needed to understand these mechanisms in detail, but one possibility is that downregulation of this pathway represents suppression of immune senescence, which can accompany T‐cell exhaustion in the tumor microenvironment.

Interestingly, there were different significant pathway associations in ovarian tumors arising in women who used low‐dose aspirin versus regular‐dose aspirin. Low‐dose aspirin (75–100 mg) is known to have antithrombotic effect, predominantly targeting COX‐1 activity in platelets,^1^ although anticoagulation pathways were not observed as being differentially regulated in the low‐dose aspirin users in our study. On the contrary, regular aspirin doses (325–1200 mg) tend to exert anti‐inflammatory effects by attenuating both COX‐1 and COX‐2 activities leading to reduce prostaglandin synthesis.^1^ While we did identify inflammatory alterations in ovarian tumors with prediagnosis regular‐dose aspirin use, prostaglandin‐related pathways were not significantly different, and the associations were stronger for extracellular matrix pathways. Notably, our results suggest that both current low‐dose aspirin use and current regular‐dose aspirin use are associated with downregulation of pathways related to metastases, but the impacted genes and biological pathways differ. Our results suggest that the canonical pathways associated with aspirin use (e.g., reduced prostaglandin synthesis, and anticoagulation) may not be the critical mechanisms underlying the potential anticarcinogenic activity of aspirin. A better understanding of cancer‐related pathways affected by aspirin use in experimental systems may identify new opportunities for therapeutic treatments for ovarian and other cancers.

To our knowledge, this is the first comprehensive RNA‐seq analysis of gene expression and biological pathways associated with prediagnosis low‐dose or regular‐dose aspirin use in well‐phenotyped, type II ovarian tumors. In the current analysis, we did not observe individual genes that were statistically significant. However, multiple biological pathways were identified, which may be due to the increased power of pathway analyses. We used three different biologic pathway databases in our analysis and observed similar pathways across databases, reducing the likelihood of false positives. However, since this analysis is based on observational studies, our study results could be influenced by the confounding effects related to the indication for aspirin use, which was not collected in these studies. While restricting to type II ovarian tumors, reducing potential effects related to different histological subtypes also reduces generalizability. Furthermore, because nearly all the women in this study were of European ancestry and were non‐Hispanic, our results may not apply to other races or ethnicities. Importantly, our study was not able to conduct experiments analyzing specific genes and pathways and therefore interpretation of the results warrant caution, and further mechanistic studies are necessary to confirm our findings.

Regardless, these findings do strongly support the need for further investigations of the effects of aspirin at different doses on tumor surveillance by the adaptive immune system and on antimetastatic pathways, which will inform novel strategies in preventing and treating ovarian cancer.

## AUTHOR CONTRIBUTIONS

**Naoko Sasamoto:** Conceptualization (supporting); data curation (equal); investigation (equal); writing – original draft (lead); writing – review and editing (lead). **Paul A. Stewart:** Data curation (equal); formal analysis (equal); investigation (equal); methodology (equal); writing – review and editing (equal). **Tianyi Wang:** Data curation (equal); investigation (equal); writing – review and editing (equal). **Zachary J. Thompson:** Investigation (equal); methodology (lead); writing – review and editing (equal). **Sean J. Yoder:** Data curation (equal); investigation (equal); writing – review and editing (equal). **Jonathan L. Hecht:** Investigation (equal); writing – review and editing (equal). **John L. Cleveland:** Investigation (equal); writing – review and editing (equal). **José R. Conejo‐Garcia:** Investigation (equal); writing – review and editing (equal). **Brooke L. Fridley:** Investigation (equal); methodology (equal); writing – review and editing (equal). **Kathryn L. Terry:** Conceptualization (equal); investigation (equal); writing – review and editing (equal). **Shelley S. Tworoger:** Conceptualization (lead); investigation (equal); writing – review and editing (equal).

## FUNDING INFORMATION

This work was supported in part by the following awards from the National Institutes of Health: P01 CA87969, U01 CA176726, UM1 CA186107, R01 CA054419, R01 CA67262, and P50 CA105009. This work was also supported in part by the Cortner‐Couch Endowed Chair for Cancer Research from the University of South Florida (to J.L.C.) and by the Biostatistics and Bioinformatics Shared Resource, Tissue Core Facility, and the Molecular Genomics Core Facility at the H. Lee Moffitt Cancer Center & Research Institute, an NCI designated Comprehensive Cancer Center (P30‐CA076292). N.S. was supported by the 2021 Scientific Scholar Award from the Marsha Rivkin Center for Ovarian Cancer Research and W81XWH2110320. The content is solely the responsibility of the authors and does not necessarily represent the official views of the National Institutes of Health.

## CONFLICT OF INTEREST STATEMENT

The authors declare no potential conflicts of interest.

## ETHICAL APPROVAL STATEMENT

The study protocol was approved by the Brigham and Women's Hospital and Harvard T.H. Chan School of Public Health Institutional Review Boards, and those of participating registries as required.

## Supporting information


Figure S1.

Figure S2.

Table S1.

Table S2.

Table S3.

Table S4.

## Data Availability

The gene expression data generated in this study have been deposited in the Gene Expression Omnibus (GEO) under accession number GSE230522. The data can be accessed at https://www.ncbi.nlm.nih.gov/geo/query/acc.cgi?acc=GSE230522. Due to appropriate adherence to ethical requirements and participant confidentiality, data used in this study are not publicly available. Further information is described at https://www.nurseshealthstudy.org/researchers (contact email: nhsaccess@channing.harvard.edu), including the procedures to obtain and access data from the Nurses' Health Studies. The NECC data that support the findings of this study are available upon request and review by study leadership.
