# Supplementary figures and images for: Associations between prediagnostic aspirin use and ovarian tumor gene expression
Source: Cancer Med. 2023 Aug 1;12(17):18405–17. doi: 10.1002/cam4.6386 (PMC10523980; doi:10.1002/cam4.6386)

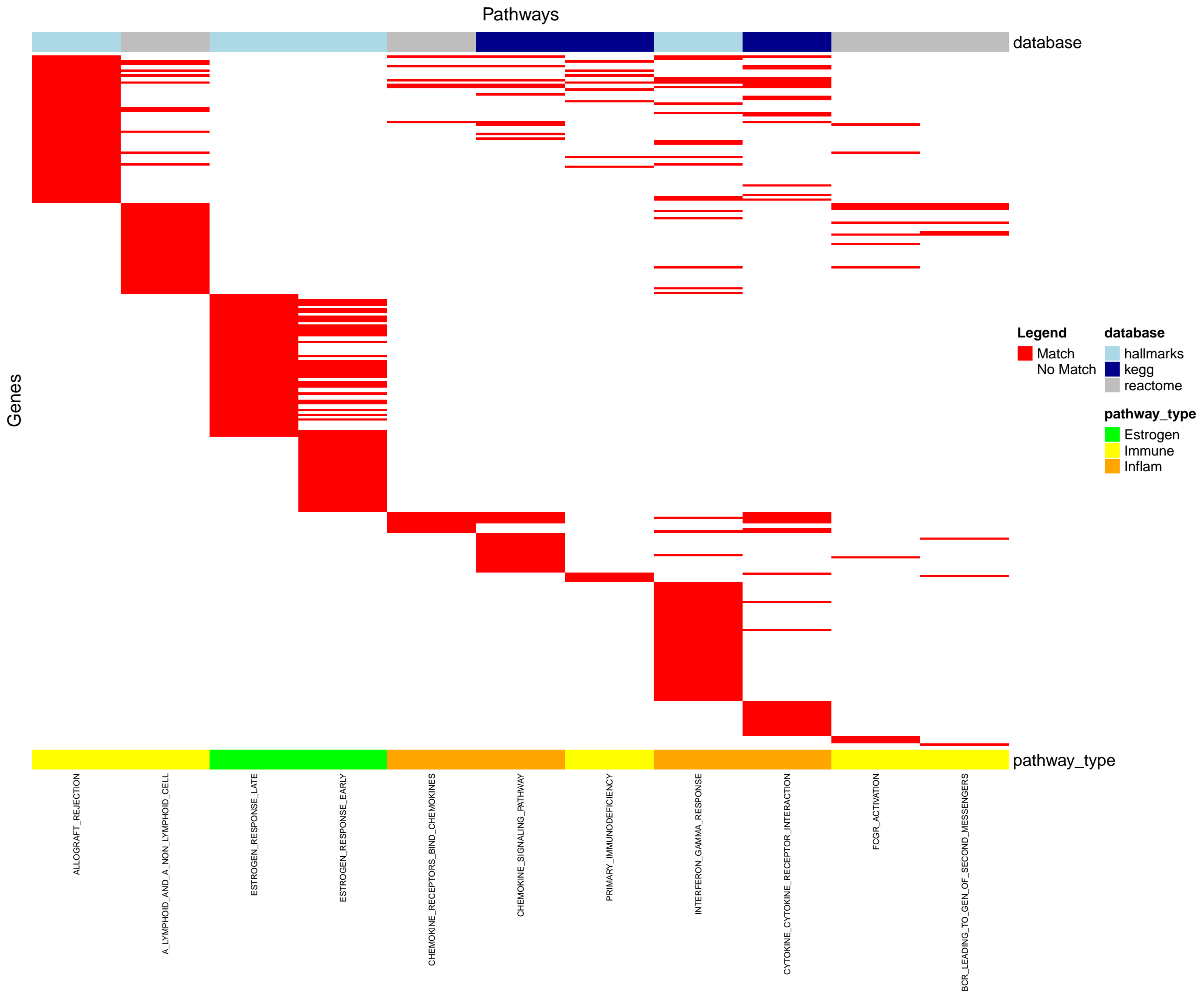

Supplement: Supplementary file 1 — Figure S1. [file CAM4-12-18405-s005.pdf]

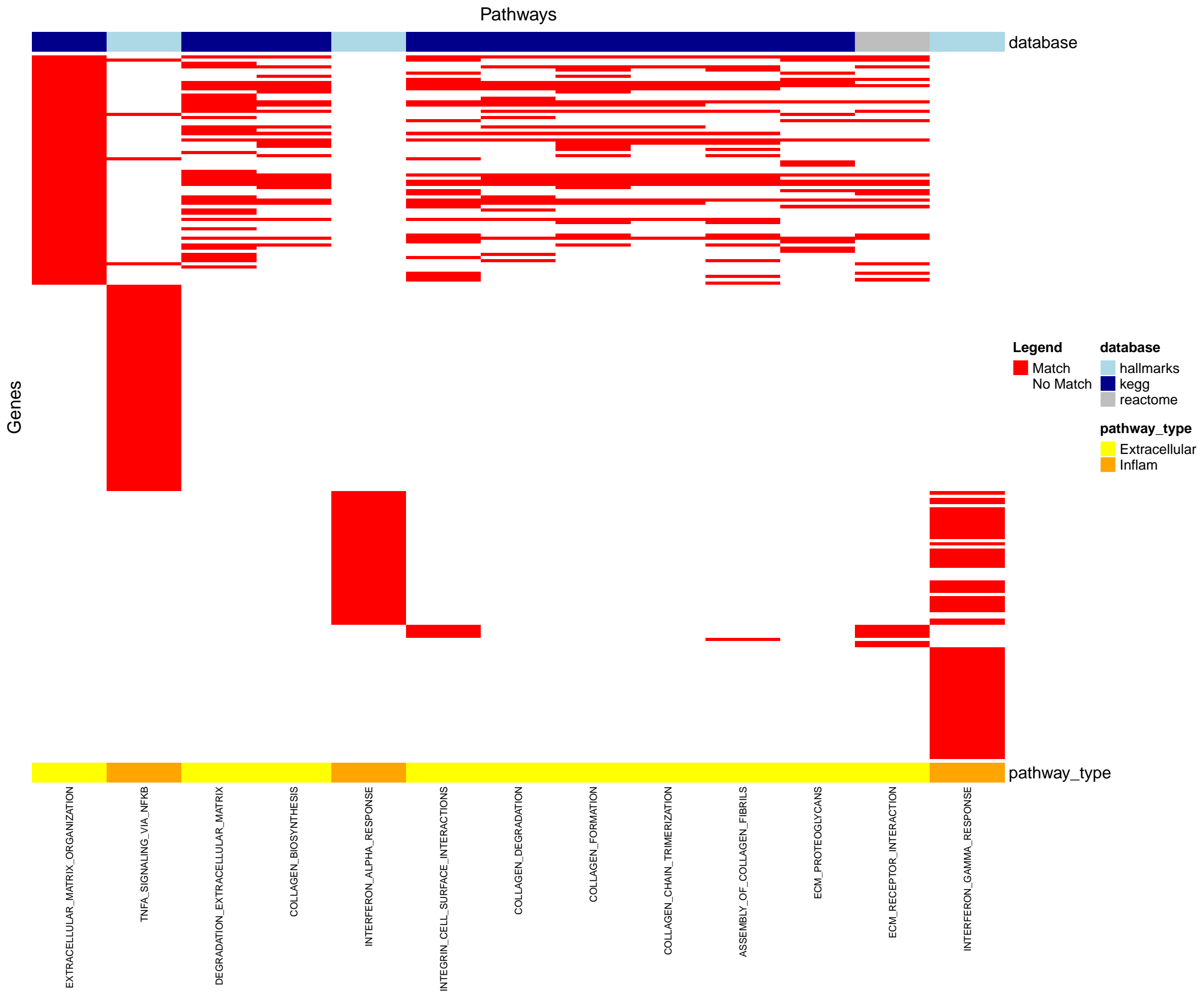

Supplement: Supplementary file 2 — Figure S2. [file CAM4-12-18405-s004.pdf]
