# Supplementary material for: Associations between prediagnostic aspirin use and ovarian tumor gene expression
Source: Cancer Med. 2023 Aug 1;12(17):18405–17. doi: 10.1002/cam4.6386 (PMC10523980; doi:10.1002/cam4.6386)
Supplement: Supplementary file 4 — Table S2. [file CAM4-12-18405-s001.docx]

**Supplementary Table S2. Significant pathways associated with current low-dose aspirin use in the 1-2 years prior to diagnosis compared to never low-dose aspirin use in type II ovarian tumor tissue^a^ in NHS, NHSII and NECC (FDR <0.01)**

| **Pathway names** | **Database** | **# of genes** | **NES** | **Unadjusted p-value^b^** | **FDR p-value** |
| --- | --- | --- | --- | --- | --- |
| **Upregulated pathways** | | | | | |
| ALLOGRAFT_REJECTION | Hallmarks of cancer | 142 | 2.25 | 1.2x10^-11^ | 5.8x10 ^-10^ |
| IMMUNOREGULATORY_INTERACTIONS_BETWEEN_A_LYMPHOID_AND_A_NON_LYMPHOID_ CELL | Reactome | 72 | 2.50 | 2.4x10^-12^ | 2.0 x10^-9^ |
| CREATION_OF_C4_AND_C2_ACTIVATORS | Reactome | 15 | 2.25 | 8.5x10^-08^ | 3.6 x10^-5^ |
| EPITHELIAL_MESENCHYMAL_TRANSITION | Hallmarks of cancer | 177 | 1.78 | 1.3x10^-05^ | 1.3 x10^-4^ |
| CELL_ADHESION_MOLECULES_CAMS | KEGG | 95 | 2.03 | 1.1x10^-06^ | 1.8 x10^-4^ |
| INTERFERON_GAMMA_RESPONSE | Hallmarks of cancer | 184 | 1.76 | 2.7 x10^-05^ | 2.0 x10^-4^ |
| INITIAL_TRIGGERING_OF_COMPLEMENT | Reactome | 20 | 2.21 | 1.1 x10^-06^ | 3.2 x10^-4^ |
| CHEMOKINE_SIGNALING_PATHWAY | KEGG | 123 | 1.92 | 7.4 x10^-06^ | 5.9 x10^-4^ |
| CHEMOKINE_RECEPTORS_BIND_CHEMOKINES | Reactome | 20 | 2.17 | 3.2 x10^-6^ | 6.9 x10^-4^ |
| PRIMARY_IMMUNODEFICIENCY | KEGG | 21 | 2.08 | 2.4 x10^-5^ | 1.3 x10^-3^ |
| CYTOKINE_CYTOKINE_RECEPTOR_INTERACTION | KEGG | 119 | 1.82 | 3.2 x10^-5^ | 1.3 x10^-3^ |
| COMPLEMENT_CASCADE | Reactome | 34 | 2.07 | 1.1 x10^-5^ | 1.7 x10^-3^ |
| BINDING_AND_UPTAKE_OF_LIGANDS_BY_SCAVENGER_RECEPTORS | Reactome | 39 | 2.07 | 1.2 x10^-5^ | 1.7 x10^-3^ |
| ANTIGEN_ACTIVATES_B_CELL_RECEPTOR_BCR_LEADING_TO_GENERATION_OF_ SECOND_MESSENGERS | Reactome | 33 | 2.04 | 1.9 x10^-5^ | 2.3 x10^-3^ |
| FCGR_ACTIVATION | Reactome | 19 | 2.07 | 3.6 x10^-5^ | 3.5 x10^-3^ |
| NEURONAL_SYSTEM | Reactome | 215 | 1.70 | 3.8 x10^-5^ | 3.5 x10^-3^ |
| SCAVENGING_OF_HEME_FROM_PLASMA | Reactome | 16 | 2.02 | 5.4 x10^-5^ | 4.2 x10^-3^ |
| HEMOSTASIS | Reactome | 449 | 1.51 | 5.6 x10^-5^ | 4.2 x10^-3^ |
| FCGR3A_MEDIATED_IL10_SYNTHESIS | Reactome | 42 | 2.01 | 5.9 x10^-5^ | 4.2 x10^-3^ |
| CELL_SURFACE_INTERACTIONS_AT_THE_VASCULAR_WALL | Reactome | 107 | 1.86 | 6.5 x10^-5^ | 4.2 x10^-3^ |
| GENERATION_OF_SECOND_MESSENGER_MOLECULES | Reactome | 28 | 2.05 | 7.5 x10^-5^ | 4.6 x10^-3^ |
| GPCR_LIGAND_BINDING | Reactome | 124 | 1.77 | 8.8 x10^-5^ | 5.0 x10^-3^ |
| PARASITE_INFECTION | Reactome | 65 | 1.88 | 1.9 x10^-4^ | 9.1 x10^-3^ |
| MUSCLE_CONTRACTION | Reactome | 121 | 1.70 | 2.2 x10^-4^ | 9.7 x10^-03^ |
| PHASE_0_RAPID_DEPOLARISATION | Reactome | 25 | 1.98 | 2.4 x10^-4^ | 9.8 x10^-3^ |
| G_ALPHA_I_SIGNALLING_EVENTS | Reactome | 187 | 1.64 | 2.6 x10^-4^ | 9.9 x10^-3^ |
| **Downregulated pathways** | | | | | |
| ESTROGEN_RESPONSE_LATE | Hallmarks of cancer | 167 | -2.15 | 2.0 x10^-9^ | 4.9 x10^-8^ |
| ESTROGEN_RESPONSE_EARLY | Hallmarks of cancer | 175 | -2.01 | 1.6 x10^-8^ | 2.6 x10^-7^ |
| GLYCOLYSIS | Hallmarks of cancer | 172 | -1.87 | 1.4 x10^-6^ | 1.7 x10^-5^ |
| P53_PATHWAY | Hallmarks of cancer | 175 | -1.68 | 2.8 x10^-5^ | 2.0 x10^-4^ |
| PROTEIN_SECRETION | Hallmarks of cancer | 94 | -1.68 | 5.2 x10^-4^ | 3.2 x10^-3^ |
| FORMATION_OF_THE_CORNIFIED_ENVELOPE | Reactome | 38 | -1.97 | 1.7 x10^-4^ | 8.7 x10^-3^ |
| KERATINIZATION | Reactome | 38 | -1.97 | 1.7 x10^-4^ | 8.7 x10^-3^ |
| HEME_METABOLISM | Hallmarks of cancer | 166 | -1.51 | 1.7 x10^-3^ | 9.6 x10^-3^ |
| NUCLEAR_EVENTS_KINASE_AND_TRANSCRIPTION_FACTOR_ACTIVATION_ | Reactome | 51 | -1.86 | 2.3 x10^-4^ | 9.7 x10^-3^ |

Abbreviations: FDR: False discovery rate; NHS: Nurses’ Health Study; NHSII: Nurses’ Health Study II; NECC: New England Case-Control Study; NES: Normalized enrichment score

^a^Type II ovarian cancer tumors include high-grade serous, poorly-differentiated, and high-grade endometrioid tumors

^b^Not adjusted for multiple testing
