# Supplementary material for: Associations between prediagnostic aspirin use and ovarian tumor gene expression
Source: Cancer Med. 2023 Aug 1;12(17):18405–17. doi: 10.1002/cam4.6386 (PMC10523980; doi:10.1002/cam4.6386)
Supplement: Supplementary file 6 — Table S4. [file CAM4-12-18405-s002.docx]

**Supplementary Table S4. Significant pathways associated with current regular-dose aspirin use in the 1-2 years prior to diagnosis compared to never regular-dose aspirin use in type II ovarian tumor tissue^a^ from participants of the NHS, NHSII and NECC (FDR <0.01) studies.**

| **Pathway names** | **Database** | **# of genes** | **NES** | **Unadjusted p-value^b^** | **FDR p-value** |
| --- | --- | --- | --- | --- | --- |
| **Upregulated pathways** | | | | | |
| INTERFERON_ALPHA_RESPONSE | Hallmarks of cancer | 92 | 2.17 | 1.1x10^-7^ | 1.4 x10^-6^ |
| INTERFERON_GAMMA_RESPONSE | Hallmarks of cancer | 184 | 1.74 | 2.5 x10^-5^ | 1.5 x10^-4^ |
| RIBOSOME | KEGG | 82 | 1.97 | 1.7 x10^-5^ | 1.7 x10^-3^ |
| EUKARYOTIC_TRANSLATION_ELONGATION | Reactome | 86 | 1.91 | 4.8 x10^-5^ | 3.1 x10^-3^ |
| SELENOAMINO_ACID_METABOLISM | Reactome | 100 | 1.84 | 8.3 x10^-5^ | 4.1 x10^-3^ |
| PD_1_SIGNALING | Reactome | 19 | 1.98 | 2.4 x10^-4^ | 1.0 x10^-2^ |
| **Downregulated pathways** | | | | | |
| EPITHELIAL_MESENCHYMAL_TRANSITION | Hallmarks of cancer | 177 | -2.41 | 1.5 x10^-14^ | 7.5 x10^-13^ |
| HYPOXIA | Hallmarks of cancer | 168 | -2.19 | 4.7 x10^-10^ | 1.2 x10^-8^ |
| TNFA_SIGNALING_VIA_NFKB | Hallmarks of cancer | 176 | -2.10 | 1.2 x10^-9^ | 2.0 x10^-8^ |
| EXTRACELLULAR_MATRIX_ORGANIZATION | Reactome | 232 | -2.17 | 5.5 x10^-11^ | 4.7 x10^-8^ |
| INTEGRIN_CELL_SURFACE_INTERACTIONS | Reactome | 72 | -2.27 | 1.6 x10^-8^ | 6.6 x10^-6^ |
| DEGRADATION_OF_THE_EXTRACELLULAR_MATRIX | Reactome | 106 | -2.22 | 2.4 x10^-8^ | 6.8 x10^-6^ |
| COLLAGEN_BIOSYNTHESIS_AND_MODIFYING_ENZYMES | Reactome | 56 | -2.32 | 8.8 x10^-8^ | 1.9 x10^-5^ |
| COLLAGEN_DEGRADATION | Reactome | 49 | -2.32 | 2.3 x10^-7^ | 3.8 x10^-5^ |
| COLLAGEN_FORMATION | Reactome | 76 | -2.22 | 2.9 x10^-7^ | 4.1 x10^-5^ |
| ANGIOGENESIS | Hallmarks of cancer | 29 | -2.20 | 4.7 x10^-6^ | 4.7 x10^-5^ |
| E2F_TARGETS | Hallmarks of cancer | 196 | -1.74 | 1.2 x10^-5^ | 9.8 x10^-5^ |
| G2M_CHECKPOINT | Hallmarks of cancer | 192 | -1.73 | 1.4 x10^-5^ | 1.0 x10^-4^ |
| COLLAGEN_CHAIN_TRIMERIZATION | Reactome | 37 | -2.24 | 2.6 x10^-6^ | 3.1 x10^-4^ |
| ASSEMBLY_OF_COLLAGEN_FIBRILS_^AND^_OTHER_MULTIMERIC_STRUCTURES | Reactome | 54 | -2.15 | 4.0 x10^-6^ | 4.2 x10^-4^ |
| ECM_PROTEOGLYCANS | Reactome | 60 | -2.09 | 9.0 x10^-6^ | 8.4 x10^-4^ |
| ECM_RECEPTOR_INTERACTION | KEGG | 71 | -2.01 | 2.2 x10^-5^ | 1.7 x10^-3^ |
| GOLGI_TO_ER_RETROGRADE_TRANSPORT | Reactome | 117 | -1.86 | 2.5 x10^-5^ | 2.1 x10^-3^ |
| SIGNALING_BY_PDGF | Reactome | 54 | -2.00 | 4.9 x10^-5^ | 3.1 x10^-3^ |
| RESOLUTION_OF_SISTER_CHROMATID_COHESION | Reactome | 114 | -1.83 | 5.3 x10^-5^ | 3.1 x10^-3^ |
| TP53_REGULATES_TRANSCRIPTION_OF_CELL_CYCLE_GENES | Reactome | 49 | -2.02 | 5.4 x10^-5^ | 3.1 x10^-3^ |
| COPI_DEPENDENT_GOLGI_TO_ER_RETROGRADE_TRAFFIC | Reactome | 84 | -1.91 | 5.5 x10^-5^ | 3.1 x10^-3^ |
| HEMOSTASIS | Reactome | 449 | -1.49 | 8.2 x10^-5^ | 4.1 x10^-3^ |
| KINESINS | Reactome | 45 | -1.94 | 1.3 x10^-4^ | 6.1 x10^-3^ |
| MITOTIC_SPINDLE | Hallmarks of cancer | 196 | -1.51 | 1.1 x10^-3^ | 6.3 x10^-3^ |
| KRAS_SIGNALING_UP | Hallmarks of cancer | 164 | -1.52 | 1.3 x10^-3^ | 6.5 x10^-3^ |
| NCAM_SIGNALING_FOR_NEURITE_OUT_GROWTH | Reactome | 47 | -1.96 | 1.6 x10^-4^ | 7.3 x10^-3^ |
| COAGULATION | Hallmarks of cancer | 95 | -1.64 | 1.7 x10^-3^ | 7.8 x10^-3^ |
| UV_RESPONSE_UP | Hallmarks of cancer | 134 | -1.54 | 2.2 x10^-3^ | 9.1 x10^-3^ |

Abbreviations: FDR: False discovery rate; NHS: Nurses’ Health Study; NHSII: Nurses’ Health Study II; NECC: New England Case-Control Study; NES: Normalized enrichment score

^a^Type II ovarian cancer tumors include high-grade serous, poorly-differentiated, and high-grade endometrioid tumors

^b^Not adjusted for multiple testing
